# Implementation of antiretroviral therapy for life in pregnant/breastfeeding HIV+ women (Option B+) alongside rollout and changing guidelines for ART initiation in rural Zimbabwe: the Lablite Project experience

**DOI:** 10.1097/QAI.0000000000001267

**Published:** 2017-04-15

**Authors:** Deborah Ford, Margaret Muzambi, Misheck J Nkhata, George Abongomera, Sarah Joseph, Makosonke Ndlovu, Travor Mabugu, Caroline Grundy, Adrienne K Chan, Fabian Cataldo, Cissy Kityo, Janet Seeley, Elly Katabira, Charles F Gilks, Andrew Reid, James Hakim, Diana M Gibb

**Affiliations:** 1 MRC Clinical Trials Unit at UCL, London, U.K.; 2 University of Zimbabwe, Harare, Zimbabwe; 3 Dignitas International, Zomba, Malawi; 4 Joint Clinical Research Centre, Kampala, Uganda; 5 Division of Infectious Diseases, Department of Medicine, Sunnybrook Health Sciences Centre, University of Toronto, Canada; 6 MRC/UVRI Uganda Research Unit of AIDS, Entebbe, Uganda; 7 Infectious Diseases Institute, Makerere University, Mulago, Uganda; 8 School of Population Health, University of Queensland, Australia

**Keywords:** antiretroviral therapy, Option B+, CD4-threshold, decentralization, primary healthcare

## Abstract

**Background:**

Life-long ART for pregnant and breastfeeding women (Option B+) was rolled-out in Zimbabwe from 2014 with simultaneous raising of the CD4 treatment-threshold in non-pregnant/breastfeeding adults and children >5 years to 500 cells/mm^3^.

**Methods:**

Lablite is an implementation project in Zimbabwe, Malawi and Uganda evaluating ART rollout. Routine patient-level data were collected for 6 months prior to and 12 months after Option B+ rollout at a district hospital and three primary care facilities in Zimbabwe (two with outreach ART; one with no ART provision prior to Option B+).

**Results:**

Between September 2013-February 2015 there were 1,686 ART initiations in the four facilities; 91% adults and 9% children aged <15 years. In the three facilities with established ART, initiations rose from 300 during 6 months before Option B+ to 869 (2.9-fold) and 463 (1.5-fold) respectively 0-6 months and 6-12 months after Option B+. Post Option B+, an estimated 43% of pregnant/breastfeeding women needed ART for their own health, based on WHO stage 3/4 or CD4≤350 (64% for CD4≤500). 74 (22%) men and 123 (34%) non-pregnant/breastfeeding women initiated ART with CD4>350 after the CD4-threshold increase. Estimated 12-month retention on ART was 79% (69%-87%) in Option B+ women (significantly lower in younger women, p=0.01), versus 93% (91%-95%) in other adults (difference p<0.001).

**Conclusions:**

There were increased ART initiations in all patient groups following implementation of WHO 2013 guidelines. Retention of Option B+ women was poorer than retention of other adults; younger women require attention as they are more likely to disengage from care.

## Introduction

WHO 2013 guidelines promoted expanded eligibility for ART by raising the CD4 threshold for treatment initiation from 350 to 500 cells/mm^3^ for HIV-infected individuals aged 5 years and over; ART was recommended for all children below 5 years; Option B (ART during pregnancy and breastfeeding) or Option B+ (provision of ART for life for pregnant/breastfeeding women) for high fertility/high HIV prevalence countries was recommended over Option A (ART perinatally only) for PMTCT. 1 The PROMISE trial subsequently confirmed superiority of Option B over Option A for reducing MTCT. 2 Malawi was the first country to implement ‘Option B+’ in September 2011. 3 By 2015, most low/middle-income countries were piloting, rolling-out or implementing Option B+. 4 Advantages of Option B+ over previous PMTCT Option A and Option B (ART during pregnancy and breastfeeding) include: simplification of PMTCT program requirements (notably no requirement for a CD4 count result to be returned before ART initiation); extended protection from MTCT in future pregnancies from conception; prevention against sexual transmission to serodiscordant partners; benefit to the woman’s health of earlier treatment and avoiding the risks of stopping and starting ART; and a simple message to communities that once ART is started, it is taken for life. 5

Striking increases in pregnant women accessing ART have been seen in countries implementing Option B+, although the first countries to implement Option B+ tended to have low PMTCT coverage. 6,7 Because Option B+ is relatively new, there are few reports of outcomes from National programmes. In the first National cohort of Option B+ women in Malawi retention at 12 months, when most African women are still breastfeeding, was only 77%; 7 retention within more recent cohorts is similar, 8 emphasizing the need for support measures for this population. At Elizabeth Glazer Pediatric AIDS Foundation sites in Uganda early 6 month data suggests slightly higher retention. 6

Zimbabwe adopted Option B+ after a National Stakeholder Consultation in 2013 9 and began piloting in July 2013 with roll-out in 2014. National guidelines issued at the end of 2013 also included recommendations to treat all HIV-infected children below 5 years and to treat older children and adults with WHO stage 1/2 disease and CD4 count <500. 10 Prior to the introduction of Option B+, the PMTCT program (providing Option A) in Zimbabwe was functioning comparatively well (78% ART coverage in pregnant women in 2013 11), population HIV and CD4 testing levels were high, and ART coverage was also high at 77% in treatment-eligible adults in 2013 12), although considerably lower in children at 46%. 12

However, ART clients were frequently travelling long distances to access treatment and ART provision in primary care (where available) was principally through outreach teams visiting from local hospitals. Implementation of Option B+ necessitated integration of ART into all PMTCT settings where services are mostly delivered by nurses at the primary health facility level; for Zimbabwe this required expanding the ART program from 1006 to 1560 facilities between 2013 and 2015. 9

The Lablite Project 13 worked with Ministries of Health in Malawi, Uganda and Zimbabwe to evaluate roll-out of ART at four non-research sites (each comprising a district hospital and 2-4 linked rural primary care facilities). In Zimbabwe, Lablite was present in Zvimba, a rural district west of Harare, with 270,000 inhabitants and estimated HIV prevalence of 14%. The district includes 2 secondary-care facilities and 31 primary care facilities. Here we report on ART provision during the 6 months prior to implementation of Option B+ (March 2014) and for the following 12 months, in one of the district hospitals and three primary care facilities. The CD4 threshold for starting ART was raised from 350 to 500 cells/mm3 in January 2014, two months earlier than implementation of Option B+, but in practice few initiations at higher CD4s occurred before Option B+ was rolled-out. Aims of this paper were to describe the impact of Option B+ provision and the increase in CD4 initiation threshold on ART provision and to quantify retention on ART, identifying predictors of high retention in care.

## Methods

The Lablite facilities were selected in consultation with Ministry of Health and Child Welfare (MOHCW). A “hub-spoke” approach was taken with Banket District Hospital being the hub for three primary care facilities; prior to the Lablite project two facilities (Nyabira, Mutorashanga) were benefiting from ART provision by Banket District Hospital outreach teams 2-weekly and one facility (Zowa) had no ART provision. In collaboration with MOHCW, healthcare workers were trained and mentored on site using national training materials, supplemented by a checklist for monitoring patients on ART and a handbook. Staff at Banket District Hospital were trained first and they mentored staff in the primary care facilities. The primary care facilities then received accreditation as ART-initiating and follow-up sites.

Routine individual patient-level data were collected regularly from ART registers, appointment registers and facility patient cards for all patients newly registered on ART between September 2013 and February 2015 by a data capturer independent from clinic staff. Registrations include patients who are initiating ART and patients described as “transfers-in” who have started ART at another healthcare facility and are moving facility for follow-up on ART (during this study, often due to primary care facilities now providing full ART services, when they had not previously). Defaulters (no visit for 90 days) were cross-checked with clinic defaulter lists (lists the nurses are required to keep within each clinic to identify defaulters for MOHCW reporting). Data were entered into bespoke database tools that mimicked the clinic paper-based tools.

Reasons for starting ART were coded for analysis in a hierarchical manner as follows: children <15 years, adults with WHO stage 3/4 disease (including tuberculosis), pregnant post-Option B+, breastfeeding post-Option B+, CD4<threshold. Pregnancy and breastfeeding were coded as reasons for initiation above CD4 for two reasons: (1) Option B+ women started ART immediately without waiting for CD4 results; and (2) not all pregnant/breastfeeding women have CD4 results. Current outcome at last data collection (between March and June 2015) was used to estimate retention on ART at end of follow-up, defined as 28 February 2015 (gaps in care prior to this were ignored). Patients who transferred-out to a new health facility were censored at the date of transfer.

Comparisons of initiation characteristics were planned between 6-month time periods, pre Option B+ and 0-6 and 7-12 months post Option B+. Retention in care was estimated separately for new ART initiations and patients who transferred-in on ART. Comparisons of retention on ART were planned between facilities and for new ART initiations between Option B+ women and other adults. Proportions were compared using chi-square tests. Rank sum tests were used to compare distributions of continuous variables. Retention in care at 6 and 12 months was estimated by the Kaplan-Meier survivor function. Hazard ratios (HRs) were estimated using Cox regression; HRs and p-values presented in the text to assess differences in retention by patient-level characteristics and facility are from univariable models (log rank tests gave similar p-values, data not shown). Multivariable Cox models were selected by backwards selection with significance level 0.1; continuous variables were included as fractional polynomials. In patients who started ART for WHO 3/4 disease or CD4<threshold we fitted separate models for the risks of death and default including all the predictors identified for the combined endpoint (cause specific HRs are presented). Cumulative incidence of death and default were estimated using a competing risks framework.^14^ STATA version 13.1 was used.

## Role of the funding source

The study was funded by DFID, UK who had no role in study design, collection, analysis and interpretation of data, the writing of the paper or the decision to submit for publication. The corresponding author had full access to all the data.

## Results

Between September 2013 and February 2015 there were 2,088 ART registrations across the four facilities, including 1,686 (81%) new ART initiations and 402 (19%) patients already on ART who transferred in from an alternative health facility; 702 (42%) new initiations and 277 (69%) transfers-in were at the primary care facilities.

**Figure 1:**
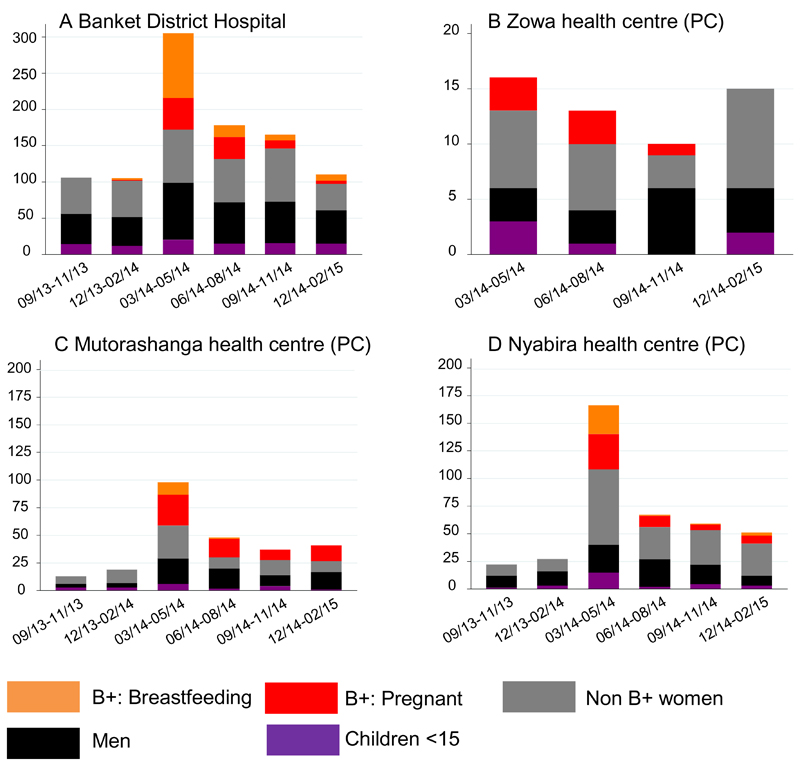
Number of new ART initiations by patient status at ART initiation, time period and health facility (PC=primary care facility)

In the three facilities with established ART provision prior to Option B+ implementation, there were 300 ART initiations in the six months prior to Option B+ roll-out compared to 869 and 463 initiations in the 0-6 months and 6-12 months afterwards respectively, corresponding to 2.9- and 1.5-fold increases, with the largest in the first quarter (table 1, figure 1). Initiations in children <15 years comprised 36 (12%), 60 (7%) and 43 (9%) of ART initiations in the same three respective periods. Infants ≤2 years were in the minority and were seen primarily at the hospital; 37/92 (40%) children were ≤2 years at the hospital compared with 3/47 (6%) at primary care facilities (p<0.001). Throughout follow-up, adult males initiating ART were older (p<0.001) and had lower pre-ART CD4s than non-pregnant/breastfeeding females (p<0.001) (Table 1). From March 2014, the median (IQR) pre-ART CD4 rose from 143 (71-243) to 216 (104-326) in men (p<0.001) and from 192 (89-263) to 279 (161-396) in non-pregnant/breastfeeding women (p<0.001). However, even between March 2014-February 2015, 79/329 (24%) men and 47/362 (13%) non-pregnant/breastfeeding women had pre-ART CD4≤100 cells/mm^3^. There were 3 ART initiations in men at the hospital where reason given for initiation was “male partner of an Option B+ female”; although all were WHO stage 1, CD4s at initiation were 66, 313 and 470 cells/mm^3^ so they were eligible for treatment for their own health.

Over the six months prior to rollout of Option B+, 34 (24%) women initiating ART were pregnant/breastfeeding, including 3 early Option B+ initiations at the hospital; excluding these 3 and one woman who was not WHO staged, 21/30 (70%) had WHO stage 3/4 disease. In contrast, in the first quarter after Option B+ rollout, initiations rose to 269 among pregnant/breastfeeding women (corresponding to a 16-fold increase) and then reduced to 82, 42 and 42 in the subsequent 3 quarters (a 6-fold increase over the first year after Option B+). After Option B+, 59/435 (13%) of pregnant/breastfeeding women were WHO stage 3 at ART initiation and none had stage 4 disease. CD4 data were incomplete (only 190/376 (51%) with WHO stage 1/2 had a CD4 recorded); 64 (34%) stage 1/2 women had ≤350 cells/mm^3^ and 47 (25%) had 351-500 cells/mm^3^, implying that overall, 43% of pregnant/breastfeeding women would have been eligible for treatment for their own health based on WHO 2010 disease progression criteria (WHO stage 3/4 or CD4≤350 cells/mm^3^) or 64% based on WHO 2013 criteria (WHO stage 3/4 or CD4≤500 cells/mm^3^). Median age at ART initiation in pregnant/breastfeeding women was similar pre- and post- Option B+ (table 1); but the proportion of adolescents <20 years increased from 1 (3%) before Option B+ to 25 (7%) in the first six months and 13 (15%) in the second six months post Option B+ (trend p=0.008).

In Zowa, a primary care facility, where ART provision was started later and alongside Option B+ rollout (from April 2014), there were 54 ART initiations, including 6 in children <15 years (figure 1).

**Figure 2:**
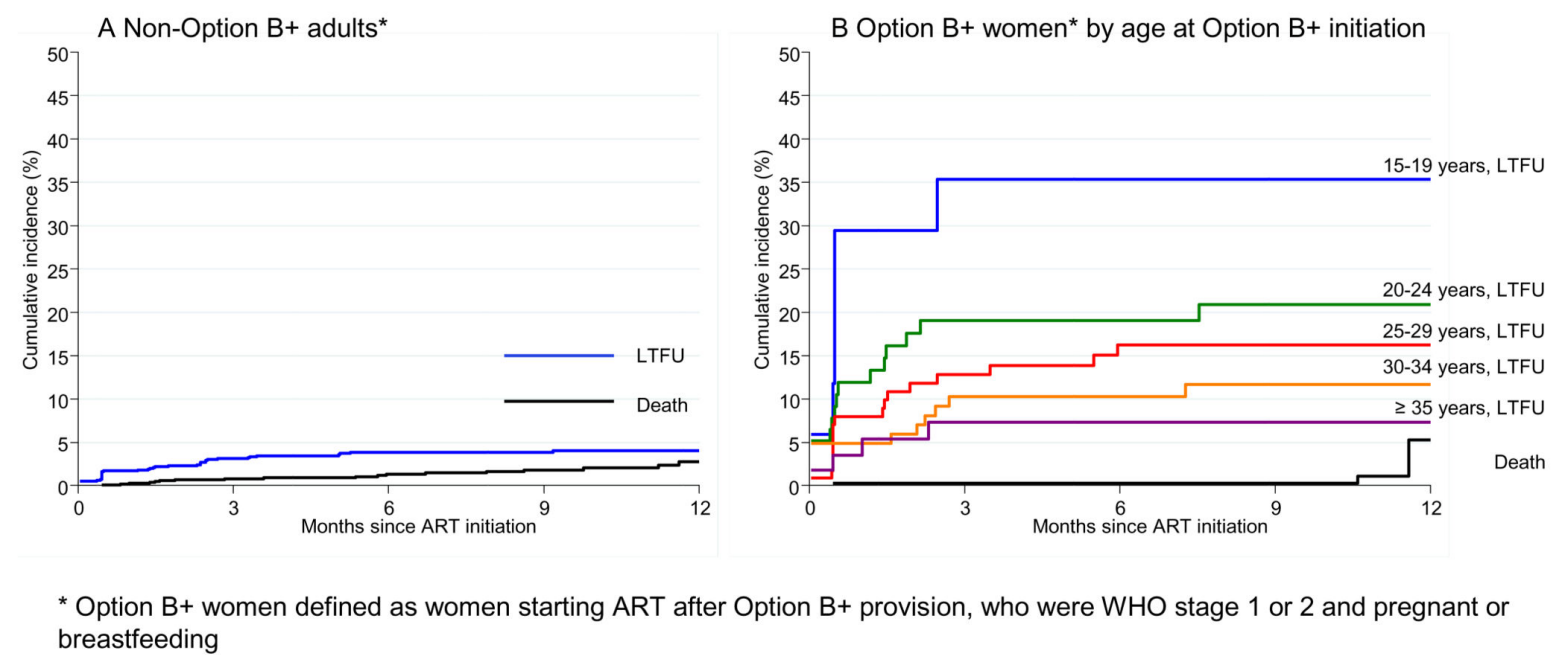
Cumulative incidence of death or default (LTFU) by time since ART initiation

Median follow-up in individuals newly initiating ART was 8 (3-11) months. Retention on ART in 1,140 adults who started ART for disease progression (all patients pre-Option B+ implementation; pregnant/breastfeeding women with WHO stage 3/4 disease and non-pregnant/breastfeeding patients post Option B+ implementation) was 95% (95% CI 93%-96%) at 6 months including 12 deaths and 39 losses to follow-up (LTFU); 12 month retention was 93% (91%-95%) (18 deaths, 40 LTFU). Figure 2a shows the cumulative incidence of death and default across all sites. Retention varied between sites (Table 2; p<0.001); 12 month retention in the primary care facilities was 86% (80%-90%) compared with 98% (96%-99%) at the hospital. There was no evidence that retention-in-care was worse when ART was initiated after Option B+ had rolled out compared with before Option B+ (Table 2). Retention was higher in patients with higher CD4 and lower in pregnant/breastfeeding patients (pre-Option B+ or also WHO 3/4 stage); when we looked at the risks of death and LTFU separately, the CD4-effect was predominantly due to an increased risk of death in patients with low CD4 whereas pregnancy/breastfeeding predicted higher LTFU but not higher mortality (table 3). Among WHO stage 1/2 patients, those with CD4>350 had similar retention to those with CD4≤350 (HR 1.11 (0.63-1.94)).

Retention on ART in 386 women who newly started ART for Option B+ was 85% (81%-88%) at 6 months (1 death, 53 LTFU) and 79% (69%-87%) at 12 months (3 deaths, 55 LTFU); this was significantly lower than retention in the adults starting ART for WHO 3/4 or CD4<threshold (across all time periods: HR 3.09 (2.15-4.46); p<0.001; restricted to March 2014 onwards: HR 4.00 (2.59-6.09); p<0.001). Retention differed markedly by age at initiation, being lower in younger women (Table 2, Figure 2b). Retention did not differ by pre-ART CD4 (Table 2). Although 32 women were LTFU by one month, 20/32 attended one post ART initiation visit (median 14 days after starting ART). Importantly, in the subgroup of women who started ART during pregnancy/breastfeeding with WHO stage 3/4 or CD4≤350 there was no evidence for worse retention after Option B+ implementation than before (HR 0.78 (0.28-2.16)).

Among 145 children <15 years at ART initiation there were 3 (2%) deaths, 16 (11%) LTFU and 10 (7%) transfers-out. The 3 children who died were all seen at the hospital; two 3 year olds died very soon after starting ART (13 days; 45 days) and one 2 year old died 12 months after initiation. At 6 months, retention on ART was 87% (80%-92%) and at 12 months it was 85% (77%-91%). We found no evidence that retention at 12 months in the primary care facilities (94% (83%-98%)) was worse than at the hospital (79% (67%-88%)).

Retention on ART in the 402 individuals who transferred into one of the facilities already on ART, was 95% (92%-97%) 6 months post transfer (0 deaths, 18 LTFU) and 94% (90%-96%) (0 deaths, 20 LTFU) at 12 months. Over half the individuals LTFU (11/20) did not return after their first registration visit.

## Discussion

The last 5 years have seen rapid changes in both PMTCT guidance and practice and CD4 threshold recommendations for ART initiation. The Lablite project 13 was working with Ministries of Health in Malawi, Zimbabwe and Uganda between 2011-2015 and collating routine data on ART rollout in non-research facilities in rural areas. In Zimbabwe, this provided an important opportunity to compare ART initiations and retention on ART before and after introduction of Option B+ and the increase in CD4 threshold.

We found that following Option B+ implementation and the increased CD4 threshold, greater numbers of all patients started ART, including children. Patients who started ART at CD4>350 or for Option B+ (with high CD4) inevitably did so as a result of guideline changes; however increases in other patient groups may have occurred anyway. Devolvement of ART care to the primary care staff with support from MOHCW allowed management of increasing patient numbers. However drug supply remains a concern; although no stock-outs of ART were reported there were anecdotal reports of patients having to return to clinic in between scheduled visits to collect ART because of low supplies, and stock-outs of cotrimoxazole did occur.

Overall mortality was low and retention in care was extremely good in patients starting ART for disease progression (93% at 12 months), better than reported for the ART programme in Zimbabwe between 2007-2010 15 and in other settings. 16 Retention was higher at the hospital than in the primary care facilities, possibly because of the Lablite model of training whereby hub staff received additional training to mentor staff at primary care facilities. Encouragingly we found no evidence that introduction of Option B+ had a detrimental effect on retention of patients starting ART for disease progression. Retention on ART was poorer in Option B+ women than in other adults starting ART, as found in Malawi, 17 but similar to retention reported in other rural districts of Zimbabwe (83%).^18^ At all four Lablite facilities, women were asked to start ART immediately; for many HIV is a new diagnosis following routine testing during antenatal care and qualitative work suggests immediate treatment may be difficult because of the limited time available to digest a positive HIV diagnosis and understand and form decisions around the uptake of a new life-long treatment.^17^,^19^

CD4 counts were obtained for 85% of men and non-pregnant/breastfeeding women prior to ART with no drop in testing after Option B+ implementation or increase in CD4 threshold. Numbers of men and non-pregnant/breastfeeding women starting ART with CD4>350 were encouraging, suggesting these individuals can be identified, although we could not distinguish individuals newly diagnosed from those already in pre-ART HIV care. Despite this, a significant proportion still start ART with low CD4s, particularly men, who are more likely to present later than women. 20,21 Around half Option B+ women had a pre-ART CD4 recorded; facilities were asked to continue providing CD4s (unlike in Malawi where CD4s are not offered to pregnant/breastfeeding women 13); it is unclear whether remaining women had no CD4 measured or whether it was not recorded. Assuming recorded CD4s were representative in Option B+ women, importantly >40% pregnant/breastfeeding needed ART for their own disease (either WHO stage 3/4 or CD4≤350), consistent with other studies. 22 It is important that these women are retained on ART for their own health as well as to prevent MTCT; healthcare workers may need focused training to support retention of Option B+ women with low CD4 counts.

Among Option B+ women, almost all attrition was recorded as LTFU; unrecorded deaths are unlikely to be a major contributor but we cannot distinguish between women who have “silently” transferred to other health facilities and those who have dropped out of care completely. Age was a clear factor in predicting loss to follow-up, with younger women more likely to be lost. This corroborates findings in Malawi 23,24 and Zimbabwe.^18^ Targeted support measures are needed for the increasing numbers of HIV-infected adolescent and young pregnant women starting ART. We were only able to look at retention on ART to 12 months and it is important to recognise that losses in this period, the majority of which were very early, are likely to have been during pregnancy or breastfeeding. In Malawi it is estimated that half of those lost in the first 6 months may never have started ART. 8 In Zimbabwe patients are asked to return to clinic 2 weeks after starting ART, which is earlier than elsewhere (including Malawi). We found that around 2/3 of women who were lost by 1 month had returned for their 2-week visit; this raises concerns that they took ART for only a short time and may now have developed resistance as the half-life of efavirenz maybe much longer than other drugs. Data from Malawi suggest that the risk of loss to follow-up is much lower after the first year; 24 however, it will be some time before comparable data are available for Zimbabwe.

In Zimbabwe since Option B+ roll-out, policy has been to encourage male partners to attend antenatal care with pregnant women and to provide them with ART, irrespective of their own CD4 count. However, this seems to have happened very rarely (only 3 men). Initiatives to engage men are critical as lack of male involvement and the complex dynamics between the women and their partners including concerns about stigma are cited by healthcare workers as a common reason for non-retention among women on Option B+. 25,26

Historically HIV-infected children have been seen in secondary and tertiary health care facilities in Zimbabwe 27 so it was encouraging to see ART initiations in under 15’s at all facilities, including the new primary care facility (where ART provision was introduced alongside Option B+). However, similar to other studies, 28 children <24 months were treated almost exclusively at the hospital, suggesting possible reluctance to test and treat infants in primary care. Retention on ART was also somewhat lower in children than in adults starting ART outside of Option B+. Previous studies of retention in care of HIV-exposed and infected children have identified a range of possible risk factors including characteristics of the carer for mortality and loss to follow-up; 29–31_ENREF_29 further research is needed to develop strategies to mitigate losses.

Following the START and TEMPRANO trials 32,33, WHO released new guidelines recommending that all HIV-infected individuals should start treatment as soon as possible after a positive diagnosis.^34^ It is unclear how quickly this recommendation will be taken up in sub-Saharan Africa. Our early data on uptake of treatment in individuals with CD4>350 following the rise in threshold suggest that there will be demand. If adopted, lessons may be learned from Option B+ roll-out; immediate treatment following provider initiated testing in “well” patients may not be dissimilar. In particular, adopting treatment for all may facilitate retention of pregnant/breastfeeding women by reducing the current disparity in access to ART between women and their partners.

Strengths of the Lablite project include the study of rural MOHCW facilities with little/no research experience and use of routine data collected from paper records. This contrasts with much of the existing Option B+ operations research which primarily focuses on larger health facilities with electronic data capture. Additionally we have data before and after the introduction of Option B+ and changes in WHO CD4 threshold guidelines and across all patient groups. The primary weaknesses include lack of information on the underlying causes of patients LTFU (common to most studies) and use of a retrospective definition of LTFU (often used in analysis of routinely-collected data where visit data may be incomplete). Our study is limited to four facilities and it remains important to collate further data to determine how generaliseable our findings are.

## Figures and Tables

**Table 1:** Characteristics at ART initiation in a District Hospital and two primary care facilities with established ART provision prior to Option B+ implementation^1^

	Pre Option B+			0-6 months post Option B+			7-12 months post Option B+			Total	
	Sep 2013-Feb 2014			Mar 2014-Aug 2014			Sep 2014-Feb 2015				
	Median (IQR)	N	% of total or of sub-group	Median (IQR)	N	% of total or of sub-group	Median (IQR)	N	% of total or of sub-group	N	% of total or of sub-group
**Total^2^**		**300**			**869**			**463**		**1632**	
											
**Children**		**36**	**12**		**60**	**7**		**43**	**9**	**139**	**9**
Age	4 (2-9)			5 (2-11)			6 (2-8)				
≤2 years		10	28		16	27		14	33	40	29
3-14 years		26	72		44	73		29	67	99	71
											
**Adult Males**		**112**	**38**		**227**	**26**		**156**	**34**	**495**	**31**
Age	38 (33,45)			37 (32,43)			37 (32,43)				
WHO stage											
Stage 1/2		19	17		68	30		35	22	122	25
Stage 3		90	81		159	70		121	78	370	75
Stage 4		2	2		0	0		0	0	2	0.4
CD4^3^	143 (71,243)			215 (103,341)			216 (112,321)				
CD4≤100		29	36		46	25		33	23	108	26
CD4 101-200		27	34		38	20		31	22	96	23
CD4 201-350		22	28		59	32		48	34	129	32
CD4 351-500		2	3		38	20		28	20	68	17
CD4>500		0	0		5	3		3	2	8	2
											
**Non-pregnant & non-breastfeeding adult females**		**110**	**38**		**224**	**26**		**180**	**39**	**514**	**32**
Age	35 (29,42)			34 (30,40)			34 (28,42)				
WHO stage											
Stage 1/2		20	18		66	29		46	26	132	26
Stage 3		88	81		157	70		132	73	377	73
Stage 4		1	1		1	0.5		2	1	4	1
CD4^4^	192 (89,263)			295 (172,398)			266 (152,395)				
CD4≤100		24	28		20	10		27	16	71	16
CD4 101-200		21	24		43	22		41	24	105	23
CD4 201-350		38	44		63	33		45	26	146	33
CD4 351-500		4	5		62	32		54	32	120	27
CD4>500		0	0		4	2		3	2	7	2
											
**Pregnant or breastfeeding adult females**		**34**	**12**		**351**	**41**		**84**	**18**	**469**	**29**
											
Age	28 (26,31)			29 (24,33)			26 (21,32)				
Pregnant		18	53		182	52		57	68	257	55
WHO stage											
Stage 1/2		12	36		305	87		71	85	388	83
Stage 3		20	61		46	13		13	16	79	17
Stage 4		1	3		0	0		0	0	1	0.2
CD4^5^	250 (148,307)			454 (304,613)			339 (245,501)				
CD4≤100		5	18		12	7		3	8	20	8
CD4 101-200		6	21		16	9		4	11	26	11
CD4 201-350		17	61		29	19		12	32	58	24
CD4 351-500		0	0		44	26		8	22	52	22
CD4>500		0	0		71	41		10	27	81	34

1 Zowa primary care health facility is not included as there was no ART provision prior to Option B+, hence its inclusion post Option B+ could distort comparisons

2 15 individuals with missing age excluded from sub-groups below

3 CD4 at initiation was available in 80 (71%), 186 (82%), 143 (92%) males by time period

4 CD4 at initiation was available in 87 (79%), 192 (86%), 167 (93%) non-pregnant & non-breastfeeding females by time period

5 CD4 at initiation was available in 28 (82%), 172 (49%), 37 (44%) pregnant or breastfeeding females by time period

**Table 2:** Predictors of death and loss to follow-up from care in adults newly starting ART

	Initiated for WHO stage 3/4 or CD4<threshold				Initiated for Option B+			
	Univariable analysis		Multivariable analysis*		Univariable analysis		Multivariable analysis*	
	N=1,140 (58 failures)^1^		N=904 (41 failures)^2^		N=386 (58 failures)^3^		N=386 (58 failures)	
	HR (95% CI)	p	HR (96% CI)	p	HR (95% CI)	p	HR (95% CI)	p
**Site**								
Hospital	1.00		1.00		1.00			
Nyabira (PC)	7.52 (3.85-14.7)		7.32 (2.96-18.1)		1.12 (0.60-2.09)			
Mutorashanga (PC)	3.74 (1.90-7.36)		4.02 (1.72-9.41)		0.61 (0.29-1.28)			
Zowa (PC)	1.38 (0.18-10.5)	<.001	-	<.001	-	0.33		
**Initiation period**								
Sep 2013-Feb 2014	1.0		1.00		-			
Mar 2014-Feb 2015	0.51 (0.30-0.87)	0.01	0.42 (0.21-0.86)	0.02	-			
**Sex**								
Male	1.00				-			
Female	0.92 (0.55-1.55)	0.77			-			
**Age at initiation (per year increase)**	0.97 (0.95-1.00)	0.07			0.94 (0.90-0.98)	0.01	0.94 (0.90-0.98)	0.01
**WHO stage at initiation**								
WHO 1/2	1.00		1.00		-			
WHO 3	0.55 (0.32-0.95)		0.56 (0.27-1.18)		-			
WHO 4	4.35 (1.02-18.6)	0.004	4.95 (0.99-24.7)	0.01	-			
**CD4 at initiation (per 10 cell/mm^3^ increase)**	0.97 (0.95-1.00)	0.02^4^	0.97 (0.95-1.00)	0.03	1.00 (0.98-1.01)	0.67		
**Pregnant/breastfeeding**								
No/male	1.00		1.00		-			
Either pregnant or	1.98 (0.97-4.04)	0.06	2.68 (0.99-7.25)	0.05	-			
breastfeeding								
Pregnant	-				1.00			
Breastfeeding	-				0.83 (0.49-1.41)	0.49		

PC: primary care facility.

* All variables presented in the univariable analysis were considered for inclusion in the multivariable model. Continuous variables (CD4 and age) were included as fractional polynomials for model selection which was based on backwards elimination (P<0.10).

1 Due to missing data, CD4 model (N=944, failures=41); WHO stage model (N=1,137, failures=58)

2 Patients with missing CD4 or WHO stage or at Zowa (no Pre Option B+ data) were dropped in the final model

3 Due to missing data, CD4 model (N=193, failures=28); due to no failures at Zowa, site model (N=379, failures=58)

4 CD4 in the univariable model was better represented by CD4^-0.5^ but we show the linear term here for comparability with the multivariable model and for ease of interpretation.

**Table 3:** Predictors of death and loss to follow-up from care in adults newly starting ART for WHO stage 3/4 or CD4<threshold

	Death and loss to follow-up		Loss to follow-up		Death	
	N=904 (41 failures)^1^		N=904 (28 failures)^2^		N=904 (13 failures)^2^	
	HR (96% CI)	p	cHR (95% CI)	p	cHR (95% CI)	p
**Site**						
Hospital	1.00		1.00		1.00	
Nyabira (PC)	7.32 (2.96-18.1)		8.56 (3.00-24.4)		4.01 (0.23-70.4)	
Mutorashanga (PC)	4.02 (1.72-9.41)		0.97 (0.25-3.71)		41.7 (4.63-376.1)	
Zowa (PC)	-	<.001	-	<0.001	-	0.001
**Initiation period**						
Sep 2013 – Feb 2014	1.00		1.00		1.00	
Mar 2014 – Feb 2015	0.42 (0.21-0.86)	0.02	0.56 (0.23-1.36)	0.20	0.19 (0.04-0.96)	0.04
**WHO stage at initiation**						
WHO 1/2	1.00		1.00		1.00	
WHO 3	0.56 (0.27-1.18)		0.71 (0.28-1.79)		0.20 (0.04-1.04)	
WHO 4	4.95 (0.99-24.7)	0.01	7.63 (0.79-74.1)	0.07	1.75 (0.17-17.7)	0.09
**CD4 at initiation (per 10 cell/mm^3^ increase)**	0.97 (0.95-1.00)	0.03	0.99 (0.97-1.02)	0.54	0.90 (0.84-0.97)	0.003
**Pregnant/breastfeeding**						
No/male	1.00		1.00		-	
Either pregnant or breastfeeding	2.68 (0.99-7.25)	0.05	5.58 (1.82-17.1)	0.07	-3	-

PC: primary care facility. cHR: cause-specific hazard ratio

1 Model selected in multivariable analysis for combined endpoint (death and loss to follow-up), see table 2.

2 The full multivariable model was run for each competing event, censoring the alternative event.

3 There were no deaths among the pregnant and breastfeeding women.
